# Left Main Coronary Artery Hypoplasia in Elderly

**DOI:** 10.1155/2016/4156581

**Published:** 2016-03-07

**Authors:** Selma Kenar Tiryakioglu, Hakan Ozkan, Hakan Bahadir, Osman Tiryakioglu

**Affiliations:** ^1^Department of Cardiology, Bursa State Hospital, Bursa, Turkey; ^2^Department of Cardiology, Bahcesehir University, Çırağan Caddesi, Osmanpaşa Mektebi Sokak, No. 4–6, Beşiktaş, 34353 Istanbul, Turkey; ^3^Department of Cardiovascular Surgery, Bahcesehir University, Çırağan Caddesi, Osmanpaşa Mektebi Sokak, No. 4–6, Beşiktaş, 34353 Istanbul, Turkey

## Abstract

Congenital anomalies of the coronary artery causing coronary occlusive disease may be of many different types. A 67-year-old woman with no coronary risk factors was referred for coronary angiography with few months' history of angina. The patient underwent coronary angiography due to ischemic cardiac symptoms with nondiagnostic exercising test. In coronary angiography, the left main coronary artery was arising from normal anatomical position; however, left anterior descending artery and circumflex artery were hypoplastic. The treatment of patient was discussed in cardiology-cardiovascular surgery council and coronary surgery was found inappropriate due to the hypoplasia of the left coronary system entirely.

## 1. Introduction

Hypoplastic coronary artery disease (HCAD) refers to congenital underdevelopment of one or more major branches of the coronary arteries with the absence of compensatory collateral circulatory vessels. Coronary artery anomalies have an incidence of approximately 1% in the general population and one postmortem survey identified HCAD in 2.2% of the patients with coronary artery anomalies [1, 2]. Postmortem analyses of athletes were identified in less than 5% of the total patients and comprised less than 33% of the observed congenital artery anomalies. Initial presentation could be sudden cardiac death in young patients. At present, congenital anomalies of the coronary arteries are being diagnosed accurately by selective coronary angiography during life. There are seldom case presentations about coronary agenesis or hypoplasia in the literature. The case we present is interesting due to complete hypoplastic left coronary system in elderly.

## 2. Case Report

A 67-year-old woman with no coronary risk factors was referred for coronary angiography with few months' history of angina. There was not any ischemic change in electrocardiography. The echocardiography demonstrated diastolic dysfunction. Patient underwent coronary angiography due to ischemic cardiac symptoms with nondiagnostic exercising test. In coronary angiography, left main coronary artery (LMCA) was arising from normal anatomical position; however, left anterior descending artery (LAD) and circumflex artery (Cx) were hypoplastic. Right coronary artery (RCA) was arising from right sinus Valsalva and well-developed collateral system perfused the left coronary system. There was not stenosis or calcification in any coronary arteries (see Video 1 in Supplementary Material available online at http://dx.doi.org/10.1155/2016/4156581). In the case we presented, ostial LMCA agenesis was not observed; however, both LAD and Cx were uniformly narrow on the basis of its intrinsic structure and were of shorter course than normal.

**Figure 1:**
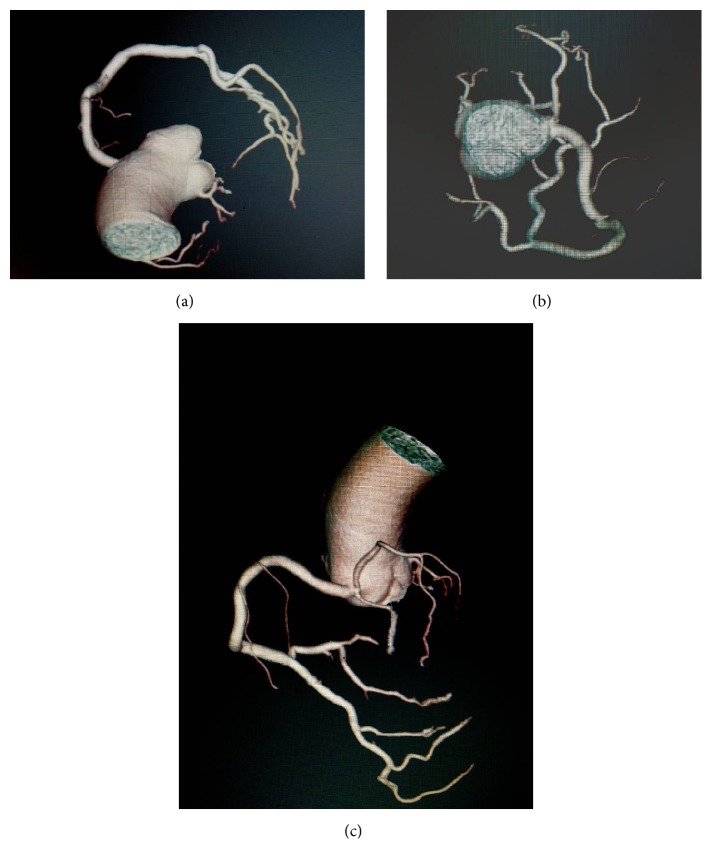
(a, b, and c) CT image of coronary artery.

The patient underwent computerized tomography (CT) for further evaluation of coronary anatomy. CT angiography confirmed the LMCA hypoplasia with well-developed collateral system from RCA as demonstrated by conventional coronary angiography (Figure 1).

The treatment of patient was discussed in cardiology-cardiovascular surgery council and coronary surgery was found inappropriate due to the hypoplasia of left coronary system entirely. We decided to stick to medical treatment including betablocker, nitrate, and statins.

## 3. Discussion

Congenital anomalies of the coronary artery causing coronary occlusive disease may be of many different types. Abnormalities of the origin of one or both arteries of the structure of arteries, their number, size, or distribution are among the possibilities [3]. Hypoplastic coronary artery or rudimentary coronary artery was described by Wenger and Chiemprapha [4, 5]. In these conditions, the coronary artery or a main coronary artery branch was uniformly narrow on the basis of its intrinsic structure and had a shorter course than normal. The LAD or LCX is usually involved, but the RCA may also be affected.

The demand of left ventricle can not be maintained in these conditions causing severe clinical presentations such as coronary occlusive disease in childhood or remaining asymptomatic. The cases with the left coronary hypoplasia and agenesis of the left main coronary ostium were presented in the literature. A case of four-month baby with shorter left coronary system which was visualized lately from RCA angiogram was presented in 1988 [6]. In our case, LMCA was hypoplastic, but the ostium agenesis was not observed. The LMCA was cannulated successfully with diagnostic catheter and angiography was performed. LAD and Cx were significantly underdeveloped. The right coronary artery was large and many collateral vessels supplied the area normally perfused by the left coronary artery. Hypoplasia of left coronary system was diffuse.

Congenital HCAD is sometimes associated with other cardiac anomalies. In 1975 and 1979, Chiemprapha and Tangchai and Line et al., respectively, published cases of aortic valve abnormalities with hypoplastic coronary artery [5].

Saji et al. demonstrated hypoplastic left coronary system with severe obstructive intimal hyperplasia in an autopsy in which a 13-year-old case was evaluated due to sudden cardiac death in 1984. Moderate interstitial fibrosis and edema have been observed. Saji et al. thought that the organization of the coronary wall and the abnormality in the maturation was responsible for the pathogenesis. In addition, they signified that HCAD has stated hosting the atherosclerotic lesions [7].

Sudden cardiac death (SCD) is a common presentation of the condition, likely from arrhythmia triggered by myocardial ischemia. In some of these cases, symptoms such as syncope, palpitations, dyspnea, and chest pain are present beforehand, while in many cases SCD is the first manifestation.

Surgical treatment for coronary artery hypoperfusion due to a single coronary artery and atresia or stenosis of the coronary ostium has been reported [6, 8]. In our case, surgery was found impossible due to the entire left coronary system hypoplasia.

In our literature surveillance, we found that most of the cases were baby or young adults presented with sudden cardiac death. McFarland et al. published a 21-year-old young male case presented with unexplained syncope. The patient underwent implantable cardioverter defibrillator (ICD) therapy for secondary prevention [2]. ICD implantation may be successful for the prevention of life-threatening cardiac arrhythmias. Heart transplantation may be indicated in patients who develop ischemic cardiomyopathy with end stage heart failure [2, 9].

In the case we presented, the patient has chronic stable angina pectoris without any symptoms of cardiac arrhythmias or syncope. The coronary anatomy was found to be inappropriate for cardiac surgery. Patient has been followed up without any cardiac events for one year.

## Supplementary Material

The video demonstrates the coronary anatomy using the right coronary angiogram.
